# TUDCA was identified as a potential therapeutic drug for nonalcoholic steatohepatitis complicated with hypercholesterolemia targeting ADRA2C by integrated bioinformatics and experimental verification

**DOI:** 10.1186/s13062-026-00926-w

**Published:** 2026-08-08

**Authors:** Hongfeng Liu, Yuxiao Fan, Zhanpeng Sun, Shuai Yuan, Biao Li, Jing Mi, Shengnan Lv, Jiajun Yang, Yihao Zhai, Qi Yuan

**Affiliations:** 1 School of Basic Medicine, Mudanjiang Medical University, Mudanjiang, 157011 China; 2 College of Life Science, Mudanjiang Medical University, Mudanjiang, 157011 China; 3 School of Basic Medicine, Qiqihar Medical University, Qiqihar, 161006 China

**Keywords:** Nonalcoholic steatohepatitis, Hypercholesterolemia, Tauroursodeoxycholic acid, ADRA2C, cGAMP-PKG signaling pathway

## Abstract

Nonalcoholic steatohepatitis (NASH) is a type of liver disease in which fat accumulates in the liver with inflammation. Hypercholesterolemia is a common autosomal dominant inherited metabolic disease. Previous studies have suggested a potential association between NASH and hypercholesterolemia, but the pathophysiological mechanisms were unclear. Bioinformatics analysis identified four core comorbidity genes (ADRA2C, IL27RA, LDB3, SF3A2). Molecular docking showed that TUDCA had the lowest binding affinity with ADRA2C and formed a stable hydrogen bond network. In vitro experiments demonstrated that TUDCA reverses PA/OA-induced high expression of ADRA2C. The cell thermal shift assay confirmed that TUDCA can directly bind to ADRA2C and enhance its thermal stability. Mechanistically, overexpression of ADRA2C significantly weakened the activation effect of TUDCA on the cGMP-PKG pathway and the recovery effect of downstream VASP phosphorylation. In vivo experiments showed that TUDCA dose-dependently improved liver function indicators infiltration in the two mice models, and inhibited the expression of core genes in liver tissue and restored the level of p-VASP protein. This research reveals the co-pathogenesis of NASH and hypercholesterolemia. These common hub genes and candidate drug may provide new targets for further mechanistic research as well as new therapies for patients with NASH and hypercholesterolemia.

**Supplementary Information:**

The online version contains supplementary material available at https://doi.org/10.1186/s13062-026-00926-w.

## Introduction

Chronic liver disease and lipid metabolism disorders have become two important problems threatening human health in the field of contemporary medical research, which have attracted wide attention around the world [1–4]. Non-alcoholic steatohepatitis (NASH) and hypercholesterolemia are typical cases of hepatic parenchymal lesions and hereditary lipid abnormalities, respectively, and their complex pathogenesis and far-reaching health implications need to be further explored [5, 6].

NASH, as an advanced and high-risk stage of non-alcoholic fatty liver disease, is spreading globally at an alarming rate as modern lifestyles are characterized by high-fat diet (HFD) and sedentary behaviors [5, 7]. Epidemiological investigations have shown that the prevalence of NASH in adults has been as high as 5% to 10% in western countries [8, 9]. NASH has also become a non-negligible health problem in Asia with the increasing incidence of metabolic syndromes such as obesity and diabetes [8]. The pathogenesis of NASH is abnormal lipid accumulation in hepatocytes, which gradually progresses to the activation of inflammatory response [10]. Inflammatory cells such as macrophages and lymphocytes infiltrate the liver parenchyma and release a large number of pro-inflammatory cytokines to cause liver cell injury [11]. As current treatments for NASH are limited, mainly through lifestyle modification through diet and exercise, there is an urgent need to elucidate potential therapeutic targets and develop effective drug therapies.

Hypercholesterolemia, as the core phenotype of dyslipidemia, encompasses a continuous disease spectrum ranging from polygenic susceptibility to monogenic inheritance. Epidemiological data shows that approximately 39% of adults worldwide have borderline hypercholesterolemia. Although familial hypercholesterolemia (FH) patients accounted for only 0.3% of the hypercholesterolemia population, they contributed approximately 20% of premature coronary heart disease events [12]. FH is a genetic disorder characterized by elevated low-density lipoprotein cholesterol levels in the blood [6, 13]. Unlike common hypercholesterolemia, FH has a definite monogenic pathogenic basis: approximately 85% of cases are caused by lost-of-function mutations in low density lipoprotein receptor (LDLR), resulting in a decrease in hepatocyte low density lipoprotein (LDL) clearance rate to 10–30% of the normal level [14]. Although the genetic basis of FH has been clarified, current research still has limitations: Firstly, the efficacy variation of existing lipid-lowering regimens for FH patients is significantly higher than that for common hypercholesterolemia; Secondly, the FH-specific biomarker system has not been established.

Despite the lack of large-scale epidemiological data directly addressing the prevalence of NASH in FH patients, both share metabolic risk factors such as obesity and insulin resistance, which may lead to elevated comorbidity rates. For example, FH patients are more likely to have liver fat deposition due to significantly elevated low-density lipoprotein cholesterol (LDL-C), and the core pathological feature of NASH is liver steatosis [15]. In addition, FH patients often have atherosclerosis, while NASH itself is an independent risk factor for cardiovascular diseases, and the coexistence of the two may exacerbate the complexity of metabolic syndrome [16, 17]. Further research on their underlying molecular mechanisms has revealed the complex connections among them. Firstly, the high LDL-C in FH patients may aggravate insulin resistance by inhibiting insulin receptor signaling [18, 19]. Insulin resistance is not only the basis for the onset of NASH but also further promotes de novo synthesis of fatty acids in the liver and steatosis [20]. Secondly, hypercholesterolemia in FH patients may interfere with mitochondrial membrane fluidity and inhibit fatty acid β-oxidation, leading to liver fat accumulation [21]. This mechanism highly overlaps with mitochondrial dysfunction in NASH, such as reduced ATP production and increased oxidative stress [22].

Bioinformatics analysis makes it possible to systematically reveal differences in gene expression profiles, alterations in key signaling pathways, and potential molecular biomarkers by integrating and analyzing large amounts of biological data [23, 24]. The use of transcriptomic techniques to compare gene expression patterns between liver tissue and normal liver in NASH patients, as well as between peripheral blood mononuclear cells in FH patients and healthy controls, is expected to accurately identify disease-specific up-regulated or down-regulated genes. These differentially expressed genes (DEGs) can be used as markers for early disease diagnosis or therapeutic targets. The rapid development of bioinformatics technology provides a new perspective and a powerful tool to reveal the mechanism of these two complex diseases. At the same time, combined with traditional animal model experiments to evaluate the efficacy of potential therapeutic drugs, thus laying a solid foundation for clinical translation.

In this study, we screened 4 hub genes (ADRA2C (α2c-adrenergic receptor), IL27RA (interleukin 27 receptor α), LDB3 (LIM domain binding protein 3) and SF3A2 (splicing factor 3 A subunit 2)) shared by NASH and FH through bioinformatics analysis. Based on molecular docking, it was found that tauroursodeoxycholic acid (TUDCA) can bind to potential biomarkers, and animal experiments further verified the therapeutic effect of TUDCA in NASH disease. This study will provide new strategies for the comprehensive prevention and treatment of these two diseases, and inject strong impetus into the field of medical research and health care.

## Materials and methods

### Microarray data collecting and processing

The NASH liver tissue microarray datasets, including GSE59045 and GSE37031, were downloaded from the Gene Expression Omnibus (GEO) database (https://www.ncbi.nlm.nih.gov/geo/). Additionally, microarray datasets of monocytes (GSE6054) and leukocytes (GSE13985) from FH patients were also obtained from GEO database. The GSE59045 dataset was generated using the GPL15207 platform, while GSE37031, GSE6054, and GSE13985 datasets were processed using the GPL570 platform. Batch correction for GEO datasets was performed using the combat function in the “SVA” package in R software (version 4.3.3) [25]. The integrated NASH expression data comprised 23 NASH samples and 7 control samples, while the integrated FH expression data consisted of 15 FH samples and 18 control samples.

### DEGs analysis

Gene symbol conversion was performed for both the NASH and FH datasets. DEGs in both the NASH and FH datasets were identified using the “limma” package in R software. DEGs were filtered based on the criteria of *p* ≤ 0.05 and |log2(fold change)| ≥ 0.585. The expression patterns of these DEGs were visualized using the “ggplot2” and “pheatmap” packages in R software, with volcano plots and heatmaps illustrating the results, respectively.

### Functional enrichment analysis

The DEGs from NASH and FH were intersected to identify shared genes, with the results visualized via a Venn diagram. Gene Ontology (GO) and the Kyoto Encyclopedia of Genes and Genomes (KEGG) analysis of the shared genes were conducted using the clusterProfiler package. The functional enrichment results were illustrated through a combination of bar plots and Sankey diagrams for a comprehensive visualization.

### Machine learning screening for hub genes

To identify hub genes associated with NASH and FH diseases, we first employed the least absolute shrinkage and selection operator (LASSO) regression algorithm. Hub genes were screened using the “glmnet” package. Subsequently, we applied recursive feature elimination in combination with the random forest (RF) algorithm for feature selection. Further refinement of the hub gene selection was performed using the “caret” package. Ultimately, hub genes associated with both NASH and FH were identified through the integration of LASSO regression and RF algorithm.

### The assessment of hub genes

Receiver operating characteristic (ROC) curves were plotted based on hub genes to evaluate the diagnostic performance of each gene in NASH and FH diseases. we performed ROC analysis using the “pROC” R package [26].

### Transcriptional regulation analysis

To predict hub gene-associated transcription factors (TFs), we performed motif enrichment analyses using the RcisTarget package. First, we import motif annotation and ranking data for the human genome from the SCENIC database. We then input gene sets (ADRA2C, IL27RA, SF3A2, and LDB3) for analysis. Motif enrichment was assessed by the cisTarget function, which calculates the normalized enrichment score (NES) to quantify the strength of the association between the gene set and each motif. We summarized the top 10 enriched motif, including key indicators such as NES, area under the curve (AUC), and the number of genes enriched in each motif. miRNA-mRNA interaction data were obtained from starBase database. To ensure that reliable interactions are screened, we set a threshold of *p* ≤ 1.0 × 10^-8 for the CLIP region. The interaction data of TFs and mRNA were obtained from JASPAR database. We used Relative Profile Score > 95% to select TFs with high mRNA specificity. The screened miRNAs, mRNAs, and TFs are imported into Cytoscape software to visualize and further analyze the miRNA-mRNA-TF regulatory network.

### Potential therapeutic drugs associated with disease

We utilized the HERB database to predict potential chemical compounds for the treatment of NASH and FH diseases. The intersection of candidate drugs for two diseases has been identified as a potential therapeutic agent. Molecular docking was subsequently performed using AutoDock Vina software to validate the interactions between hub genes and these predicted compounds. Specifically, we obtained the molecular structures of lycopene and TUDCA in SDF format from the PubChem database. These structures were converted to PDB format using OpenBabel 3.1.1, processed with AutoDockTools 1.5.7, and saved in pdbqt format. The 3D structures of the target proteins ADRA2C, IL27RA, LDB3, and SF3A2 were retrieved from the PDB database. Water and non-protein organic molecules were removed using PyMOL, hydrogen atoms were added, and the treated structures were imported into AutoDockTools 1.5.7, designated as receptors, and saved in pdbqt format. Molecular docking was performed using AutoDock Vina, and the docking results were visualized with PyMOL.

### Cell culture and treatment

The human normal liver cell line L-O2 was purchased from Rosetta Stone Biotechnology Co., Ltd., (Taiyuan, China). It was cultured in DMEM medium containing 10% fetal bovine serum and 1% penicillin/streptomycin at 37 °C in a 5% CO₂ incubator. To induce the model of liver cell lipid toxicity injury, L-O2 cells were seeded at an appropriate density and when the cell fusion rate reached 70% to 80%, the medium was replaced with a serum-free medium containing palmitic acid (PA, 250 µM) and oleic acid (OA, 500 µM) for further culture for 24 h. The TUDCA treatment group was co-incubated with the final concentrations of 3 µM or 6 µM TUDCA simultaneously when PA/OA induction was carried out. Cells were pretreated with KT-5823 (1 µM, selective PKG inhibitor) for 30 min prior to PA + OA and/or TUDCA treatment.

### Cell transfection

The ADRA2C siRNA, ADRA2C overexpression plasmid (pcDNA3.1-ADRA2C) and the empty vector control plasmid were purchased from GenePharma Co., Ltd. (Suzhou, China). L-O2 cells were seeded in 6-well plates. When the fusion rate reached 60% − 70%, they were transfected using Lipofectamine 3000 transfection reagent according to the instructions. After 48 h of transfection, the cells were collected and the overexpression efficiency was verified by western blot.

### Cellular thermal shift assay (CETSA)

L-O2 cells were treated with PA/OA and intervened with TUDCA (6 µM), washed with PBS and resuspended. They were then aliquoted into PCR tubes. Gradient heating treatment was performed on a PCR machine with temperatures set at 37 °C, 40 °C, 43 °C, 46 °C, 49 °C, 52 °C, 55 °C, and 58 °C. Each temperature point was heated for 3 min, followed by cooling at room temperature for 3 min. The cells were lysed by repeated freezing and thawing in liquid nitrogen, and the supernatant was collected for western blot to detect the residual amount of ADRA2C protein.

### Animal experiments

Male 6–8 week-old C57BL/6J mice were housed in separate ventilated cages at the specific pathogen free facility of Mudanjiang Medical University under controlled environmental conditions. All animal experiments were approved by the Animal Experiment Ethics Committee of Mudanjiang Medical University (No. IACUC20254421‑548). The mice were euthanized at the end of the experiment.

The mice were acclimated for 1 week before the experiment. In the HFD model, mice were randomly divided into 4 groups (*n* = 5). One group of mice was fed a normal control diet (NCD), while the other three were fed HFD. After 4 weeks of HFD feeding, one group of HFD-treated mice was given TUDCA at a dose of 100 mg/kg body weight once a day, and another group of HFD-treated mice was given TUDCA at a dose of 200 mg/kg body weight once a day for 2 weeks.

In the high-cholesterol diet (HCD) model, the mice were randomly divided into 4 groups (*n* = 5). One group of mice was fed NCD, while the other three were fed HCD. After 4 weeks of HCD feeding, one group of HCD-treated mice was given TUDCA at a dose of 100 mg/kg body weight once a day, and another group of HCD-treated mice was given TUDCA at a dose of 200 mg/kg body weight once a day for 2 weeks.

### Biochemical analysis

Blood samples of mice were centrifuged at 1000 × *g* for 10 min to obtain serum. Serum alanine aminotransferase (ALT), aspartate aminotransferase (AST), triglyceride (TG) and cholesterol (CHO) levels were detected using kits of Nanjing Jiancheng Bioengineering Institute (Nanjing, China). In addition, according to the instructions, the levels of intracellular TG and nonesteified fatty acid (NEFA) were detected using the kit from Nanjing Jiancheng Institute of Bioengineering (Nanjing, China).

### ELISA

Collect the cell culture supernatant or cell lysate, and follow the instructions of the cGAMP ELISA kit for operation. Measure the absorbance value at 450 nm using an enzyme reader, and calculate the cGAMP concentration based on the standard curve.

### Flow cytometry

The cells were resuspended in PBS containing BODIPY 493/503 dye solution and incubated at 37 °C in the dark for 30 min. The supernatant was removed by centrifugation, and the cells were washed twice with PBS and resuspended. The cells were also resuspended in serum-free medium containing DCFH-DA probe and incubated at 37 °C in the dark for 30 min. The supernatant was removed by centrifugation, and the cells were washed twice with PBS and resuspended. The fluorescence intensity of the above samples was detected using a flow cytometer.

### Histology and immunohistochemistry

Paraffin or cryostat sections were prepared as described previously [27]. Paraffin sections were stained with hematoxylin and eosin (H&E). Cryostat sections were stained with Oil red O. For immunohistochemistry, cryostat sections were incubated with anti-F4/80 antibody and anti-CD11b antibody (BioLegend, San Diego, CA, USA). HRP-conjugated goat anti-rat IgG was used as secondary antibody. The sections were examined under a microscope (DM3000, Leica).

### Western blot

Western blot analysis was performed as described previously [28]. Total protein was extracted from liver tissue with RIPA lysis buffer (Beyotime, Beijing, China). The protein was separated at 115 V by SDS-PAGE and transferred to PVDF membrane at 200 mA for 1 h. The membrane was filled with 5% milk powder for 1 h and incubated at 4 °C overnight with primary antibodies: anti-ADRA2C、anti-VASP、anti-p-VASP、anti-β-actin (Affinity, Cincinnati, OH, USA). Enzyme-labeled goat anti-Rabbit IgG as secondary antibody. All bands were quantified using ImageJ.

### Real-time quantitative PCR (RT-qPCR)

RT-qPCR analysis was performed as described previously [29]. Total RNA was isolated from liver tissues using Trizol reagent (Beyotime, Beijing, China), and cDNAs were synthesized using BeyoRT™ II cDNA kit (Beyotime, Beijing, China). RT-qPCR analysis was performed using the BeyoFast™ SYBR Green qPCR Mix kit (Beyotime, Beijing, China). Data were analyzed using the 2^−ΔΔCT^ method and normalized to β-actin expression. The primers used for L-O2 cells are as follows: *TNFA*, 5’-CTCTTCTGCCTGCTGCACTTTG-3’ (forward) and 5’-ATGGGCTACAGGCTTGTCACTC-3’ (reverse); *IL6*, 5’-AGACAGCCACTCACCTCTTCAG-3’ (forward) and 5’-TTCTGCCAGTGCCTCTTTGCTG-3’ (reverse); *IL1B*, 5’-CCACAGACCTTCCAGGAGAATG-3’ (forward) and 5’-GTGCAGTTCAGTGATCGTACAGG-3’ (reverse). *ACTB*, 5’- CACCATTGGCAATGAGCGGTTC-3’ (forward) and 5’-AGGTCTTTGCGGATGTCCACGT-3’ (reverse). The primers used for mice are as follows: *Adra2c*, 5’- TGCTCTTCTGCACCTCGTCCAT-3’ (forward) and 5’-CGCTTCAGGTTGTACTCTACCG-3’ (reverse); *Il27ra*, 5’- CCAACCTGTCTCTGGTGTGCTT-3’ (forward) and 5’- TACTCCAACGGTTTCCTGGTCC-3’ (reverse); *Ldb3*, 5’-ATGGCACACCACCTGCTTTGTC-3’ (forward) and 5’- ATGGCACTTGGTGCTGAACAGG-3’ (reverse). *Sf3a2*, 5’- ATGGCTGCTGAGCCCTATGAGA-3’ (forward) and 5’-GCTTGGTTTCTCTGTTCCAGTGG-3’ (reverse). *Actb*, 5’- CATTGCTGACAGGATGCAGAAGG-3’ (forward) and 5’- TGCTGGAAGGTGGACAGTGAGG-3’ (reverse).

### Statistical analysis

All data were presented as mean ± SD and analyzed using GraphPad Prism (version 9.0) and R (version 4.3.3). Student’s t test was used for variable differences between the two groups, and one-way ANOVA and multiple comparison test were used for variable differences between more than two groups. *p* < 0.05 was considered statistically significant.

## Results

### Identification of DEGs

**Fig. 1:**
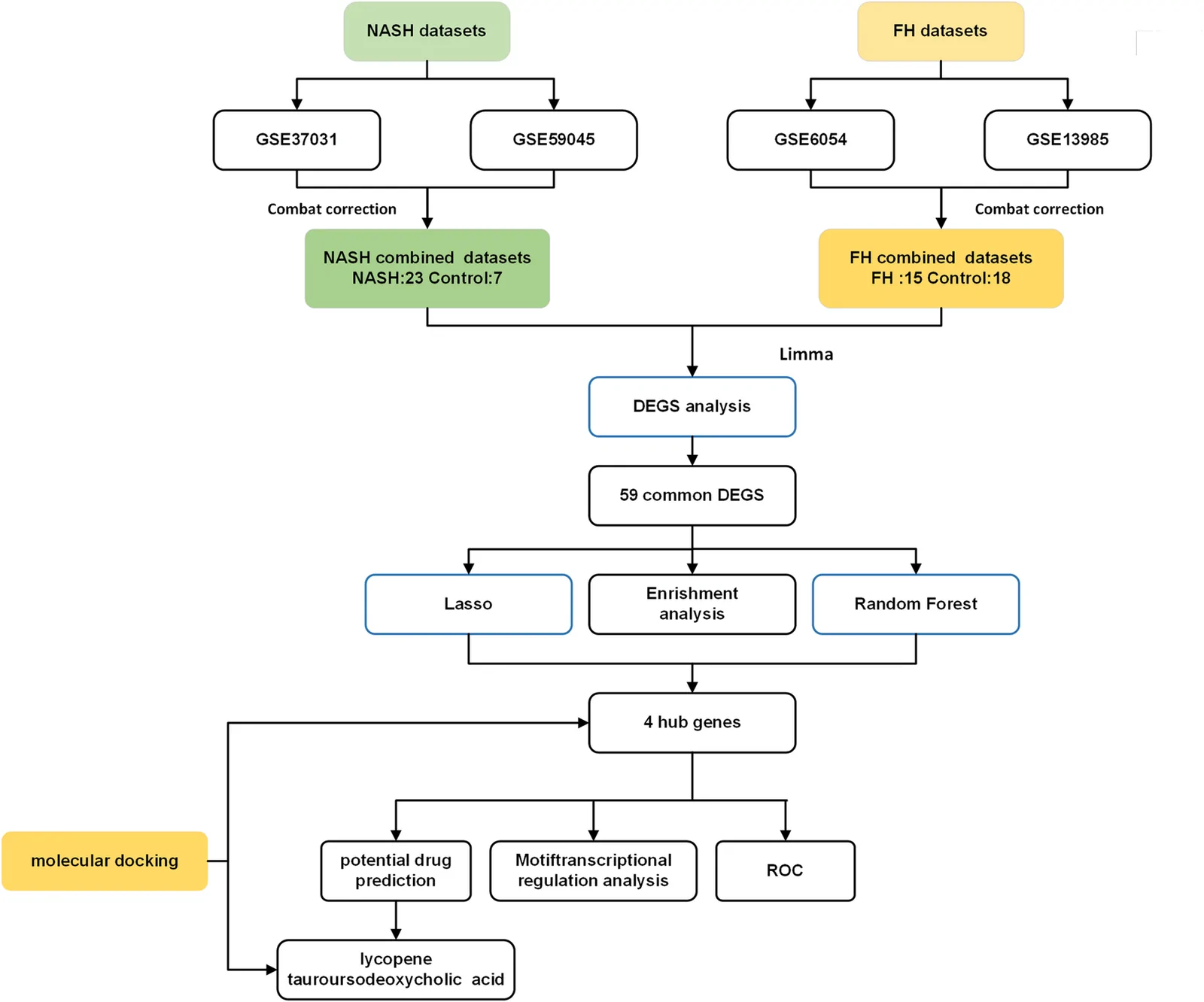
Overall schematic diagram of the study design

The bioinformatics analysis workflow is depicted in Fig. 1. Two NASH datasets and two FH datasets were retrieved from the GEO database and integrated after batch effect correction. Following normalization, the final integrated NASH dataset included 23 NASH samples and 7 control samples, while the FH dataset included 15 FH samples and 18 control samples. As shown in supplementary Figure S1, batch effect removal significantly reduced the variability between the datasets, improving their comparability in subsequent analyses.

**Fig. 2:**
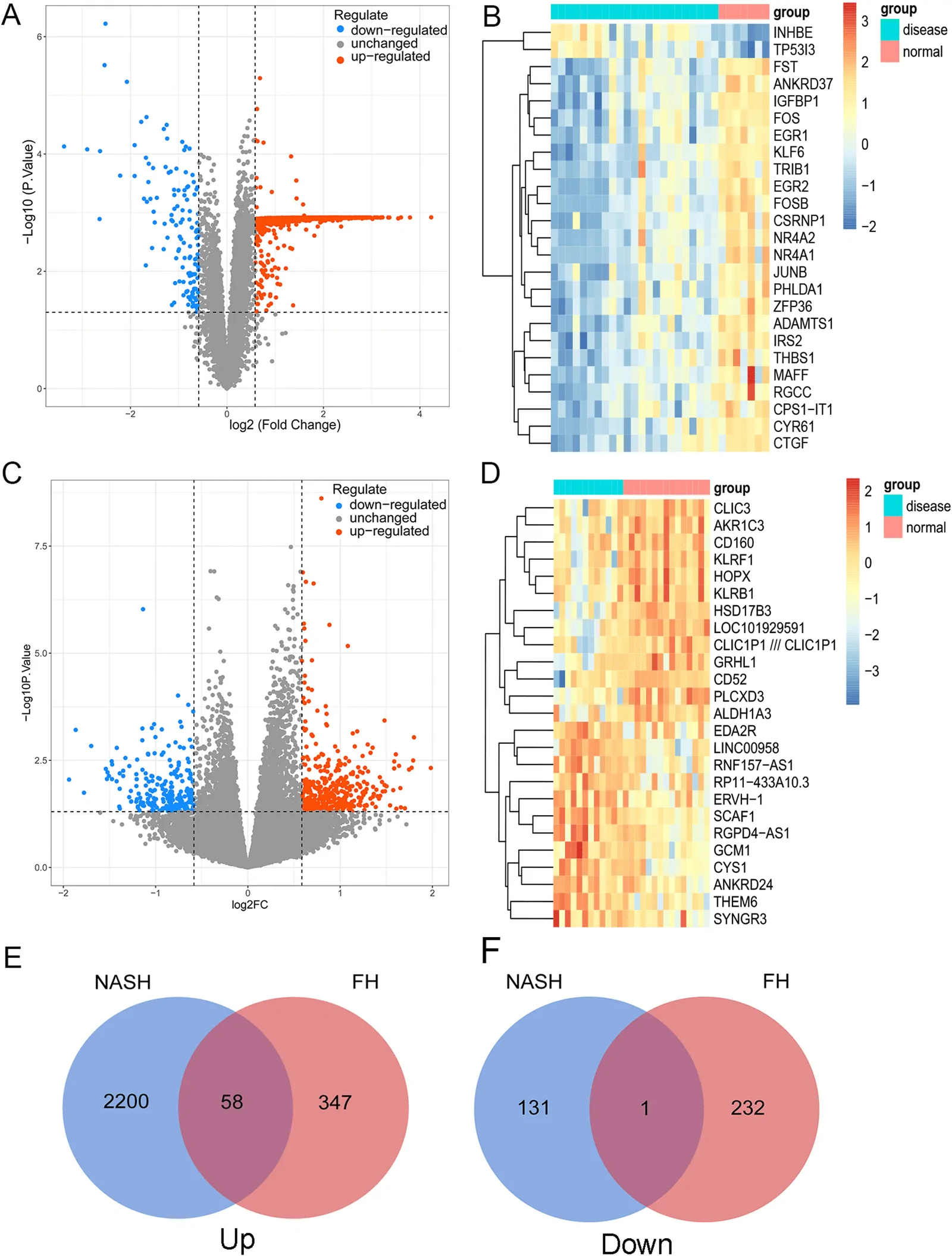
Identification of common DEGs. Volcano diagram (**A**) and heatmap (**B**) of DEGs in NASH. Volcano diagram (**C**) and heatmap (**D**) of DEGs in FH. Venn diagram identifies co-upregulated (**E**) and co-downregulated (**F**) DEGs

To explore the hub genes regulating the progression of NASH and FH, we used “limma” R package to identify DEGs for the two diseases. Compared with control samples, NASH samples had a total of 2,390 DEGs, including 2,258 up-regulated genes and 132 down-regulated genes (Fig. 2A). In addition, 638 DEGs were identified in FH samples compared to control samples, of which 405 genes were up-regulated and 233 genes were down-regulated (Fig. 2C). The heatmap presents the top 25 DEGs for NASH and FH, respectively (Fig. 2B, D). Subsequently, the intersection analysis of upregulated and down-regulated DEGs from NASH and FH resulted in 58 intersecting up-regulated DEGs and 1 intersecting down-regulated DEG (Fig. 2E, F).

#### Screening of hub genes with diagnostic value via machine learning

**Fig. 3:**
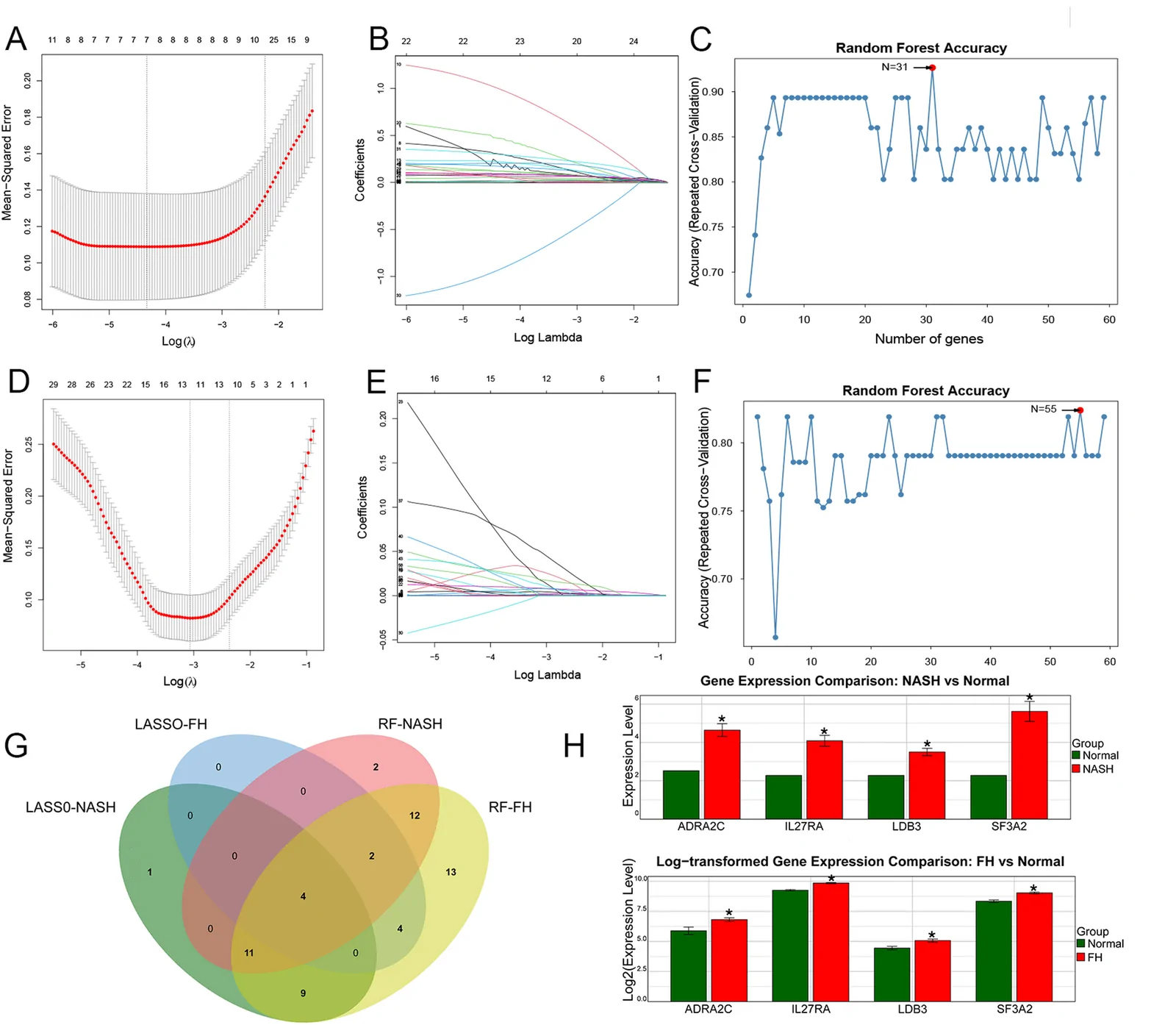
Identification of hub genes via machine learning. (**A**, **B**) The selection process of the optimum value of the parameter λ and the variation characteristics of variable coefficients in lasso regression model of NASH. (**C**) The RF algorithm of NASH. (**D**, **E**) The selection process of the optimum value of the parameter λ and the variation characteristics of variable coefficients in lasso regression model of FH. (**F**) The RF algorithm of FH. (**G**) Venn diagram of overlapping hub genes between lasso regression and RF algorithm. (**H**) Expression levels of 4 hub genes (ADRA2C, IL27RA, LDB3, and SF3A2) in NASH and FH. * *p* < 0.05

Given that common hub genes between NASH and FH may play a key role in the diagnosis of patients, we used machine learning to identify potential biomarkers. For NASH disease, we identified 25 potential candidate genes using LASSO regression algorithm (Fig. 3A, B) and 31 potential candidate genes using RF algorithm (Fig. 3C). For FH disease, we used LASSO regression algorithm to identify 10 potential candidate genes (Fig. 3D, E) and RF algorithm to identify 55 potential candidate genes (Fig. 3F). The intersection of potential candidate genes identified by machine learning for NASH and FH diseases resulted in 4 overlapping hub genes (ADRA2C, IL27RA, LDB3 and SF3A2) (Fig. 3G). The 4 hub genes were upregulated in both NASH and FH diseases (Fig. 3H).

### Transcriptional regulation analysis of hub genes

**Fig. 4:**
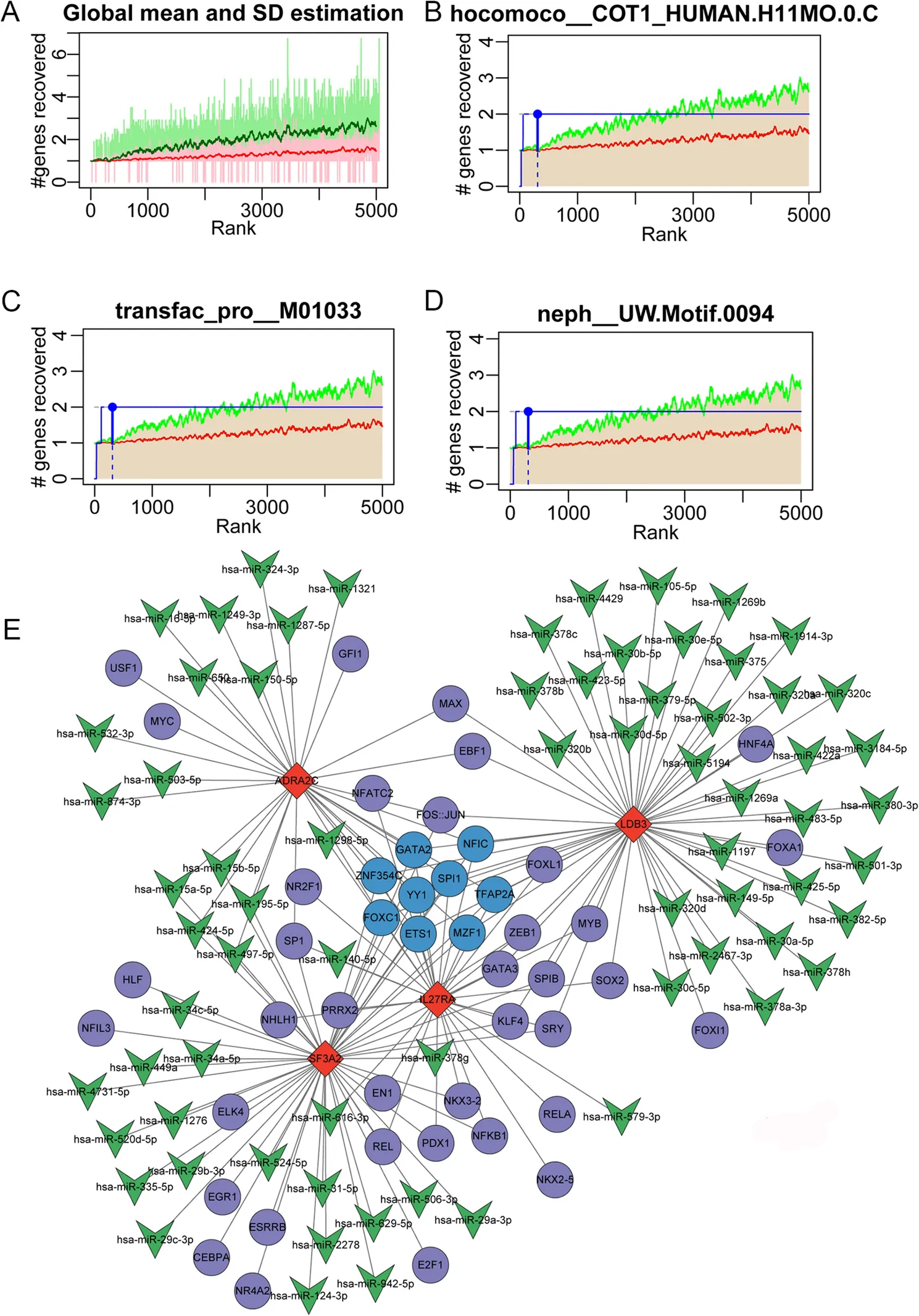
Transcriptional regulation analysis. (**A-D**) The three motifs with the highest AUC value. The red line is the mean value of the recovery curve of each motif, the green line is the mean ± SD, and the blue line is the recovery curve of the current motif. The point of maximum distance between the current motif and the green curve is the selected maximum enrichment level. (**E**) The miRNA-mRNA-TF regulatory network. Green arrows represent miRNAs, red diamonds denote mRNAs, purple circles signify TFs, and blue circle indicates the TFs shared by 4 hub genes

We found 4 hub genes controlled by other shared regulatory processes. Therefore, we hypothesized that these 4 hub genes might be regulated by the same TFs. The cumulative recovery curve, motif-TF annotation and the selection of significant genes were obtained by enrichment analysis of the TFs of the 4 hub genes (Fig. 4A-D). The results showed that the motif with the highest normalized enrichment score was hocomoco_COT1_HUMAN.H11MO.0.C, which was more common in IL27RA and LDB3 genes (supplementary Figure S2). Recent studies have highlighted that the miRNA-mRNA-TF regulatory network plays a crucial role in identifying biomarkers and understanding disease progression [30, 31]. Therefore, we further constructed a miRNA-mRNA-TF regulatory network to identify transcriptional and post-transcriptional regulators of biomarkers (Fig. 4E). Notably, 9 TFs (ZNF354C, FOXC1, GATA2, YY1, ETS1, SPI1, NFIC, MZF1, TFAP2A) were identified as exerting biological roles by co-regulating hub genes.

### Candidate drug screening and experimental verification of TUDCA targeting ADRA2C

**Fig. 5:**
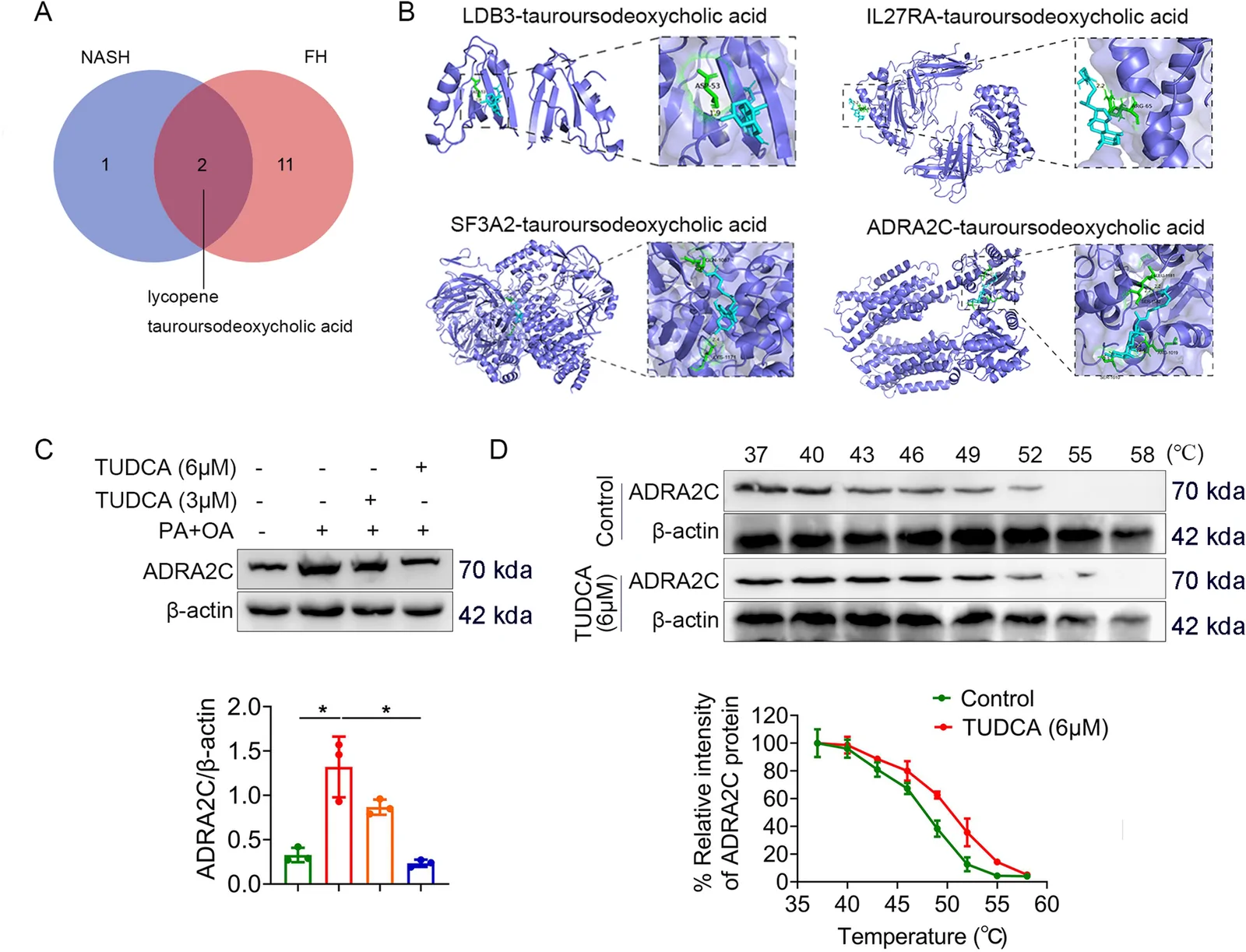
Prediction of potential therapeutic agents for NASH and FH. (**A**) Venn diagram of candidate drugs for NASH and FH disease. (**B**) Molecular docking analysis of TUDCA with ADRA2C, IL27RA, LDB3, and SF3A2. (**C**) Western blot of ADRA2C expression in PA/OA-treated hepatocytes with or without TUDCA (3 or 6 µM). (**D**) CETSA showing thermal stabilization of ADRA2C by TUDCA from 37 °C to 58 °C. *p < 0.05

We utilized the HERB database to predict potential therapeutic agents for NASH and FH diseases. The predictions were then cross-analyzed to identify two overlapping compounds, lycopene and TUDCA, as potential treatments for both diseases (Fig. 5A). We further performed molecular docking to explore the interactions between these potential drugs and hub targets ADRA2C, IL27RA, LDB3 and SF3A2. We found that lycopene did not form hydrogen bonds with these four core targets (Supplementary Figure S3). TUDCA could bind to the proteins of ADRA2C, IL27RA, LDB3, and SF3A2 through hydrogen bonds (Fig. 5B). Among these targets, ADRA2C exhibited the lowest binding energy (i.e., the most favorable binding affinity) with TUDCA. Therefore, TUDCA was selected for validation in the subsequent experiments.

The PA/OA-induced hepatocyte model demonstrated that the expression of ADRA2C protein was significantly upregulated in vitro experiments. Moreover, 6 µM TUDCA treatment could effectively reverse this trend (Fig. 5C). The CETSA experiment further confirmed that TUDCA treatment significantly enhanced the thermal stability of ADRA2C. The control group showed that the protein was almost completely degraded at 49 °C, while the treated group still detected residual protein at 52 °C and 55 °C (Fig. 5D). These results suggest that TUDCA can directly bind to ADRA2C, indicating a physical interaction between TUDCA and the ADRA2C protein.

### TUDCA improves hepatocyte lipotoxicity through the ADRA2C/cGAMP-PKG axis

**Fig. 6:**
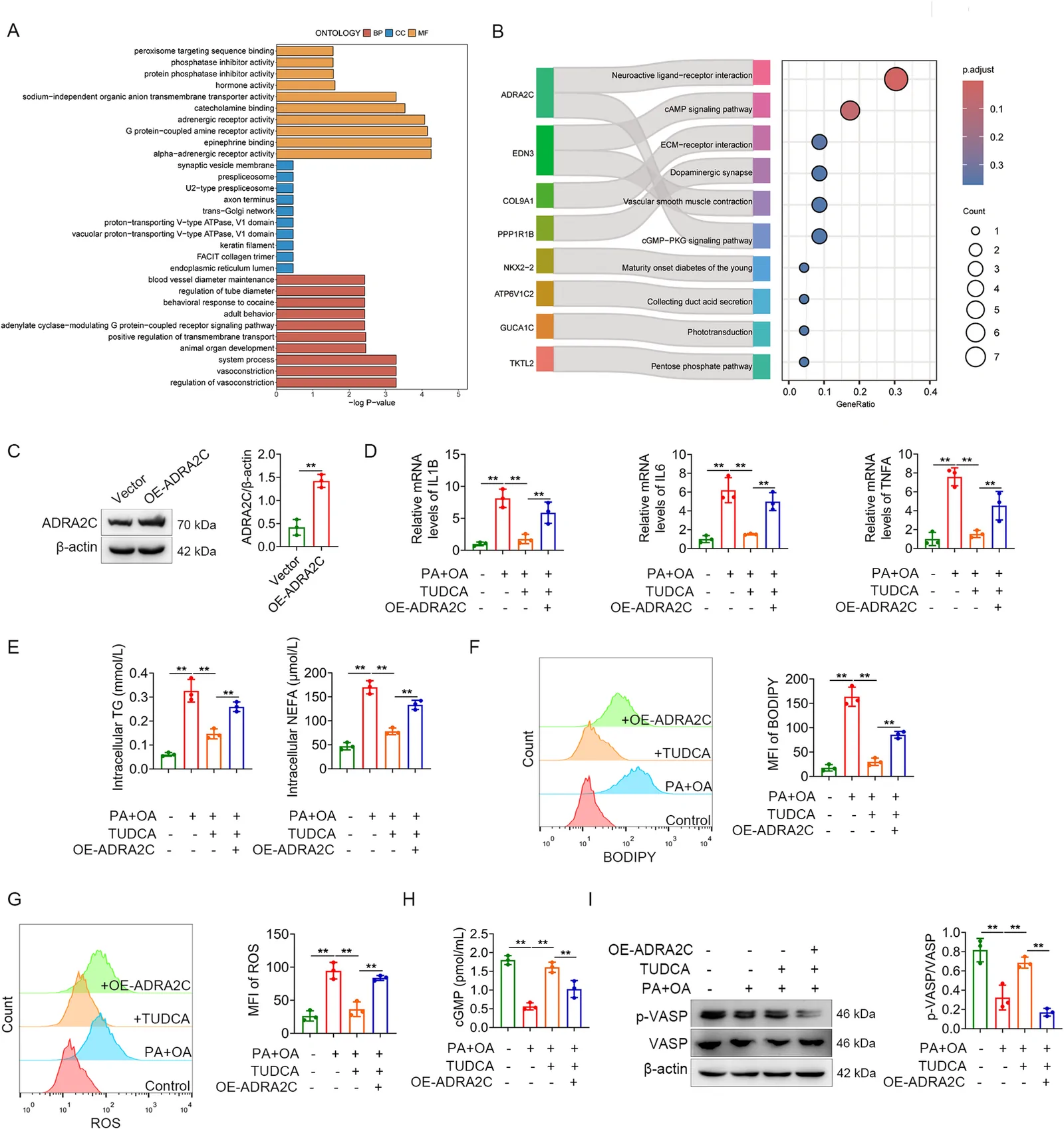
ADRA2C mediates the protective effect of TUDCA via the cGAMP-PKG signaling pathway. (**A**) GO enrichment analysis of common DEGs. (**B**) KEGG enrichment analysis of common DEGs. (**C**) Western blot validation of ADRA2C overexpression efficiency in L-O2 cell lines. L-O2 human normal hepatocytes were treated with PA/OA to induce lipotoxicity, with or without TUDCA (6 µM) treatment, and/or transfected with ADRA2C overexpression plasmid. (**D**) qRT-PCR analysis of IL-1B, IL-6, and TNFA mRNA. (**E**) Quantification of intracellular TG and NEFA content. (**F**) Flow cytometric analysis of intracellular lipid content using BODIPY 493/503 staining. (**G**) ROS level detection by flow cytometry. (**H**) ELISA-based measurement of intracellular cGAMP concentration. (**I**) Western blot analysis of total VASP and phosphorylated VASP (p-VASP) protein levels. **p < 0.01

To clarify the downstream mechanism by which ADRA2C participates in disease progression, we first conducted GO enrichment analysis and KEGG pathway annotation on the common DEGs between NASH and FH. The GO analysis revealed that, in terms of biological processes (BP), the genes were primarily enriched in regulation of vasoconstriction, and system process. No significantly enriched terms were observed in the cellular component (CC) category, as all -logP-values were below 1. For molecular functions (MF), the genes were mainly involved in α-adrenergic receptor activity, epinephrine binding, and G protein-coupled amine receptor activity (Fig. 6A). The KEGG analysis indicated that ADRA2C were mainly associated with neuroactive ligand-receptor interactions and the cGMP-PKG signaling pathway (Fig. 6B).

Based on the above analysis, we constructed ADRA2C overexpressing hepatocytes (Fig. 6C) and conducted functional rescue experiments. As shown in Fig. 6D-G, PA/OA treatment significantly induced an increase in the mRNA levels of inflammatory factors (IL-1β, IL-6, TNF-α), an increase in intracellular TG and NEFA accumulation, as well as an increase in BODIPY lipid droplet staining and reactive oxygen species (ROS) levels. TUDCA treatment could effectively reverse these pathological phenotypes; however, the overexpression of ADRA2C significantly weakened the protective effect of TUDCA, manifested as a rebound in inflammatory factors, aggravated lipid deposition, and a further increase in oxidative stress levels. Furthermore, we conducted siRNA-mediated ADRA2C knockdown experiments in PA/OA-treated hepatocytes. The results showed that knockdown of ADRA2C significantly reduced the elevated levels of TG and NEFA induced by PA/OA. However, when TUDCA was combined with ADRA2C knockdown treatment, no further decrease in TG or NEFA levels was observed, indicating that the lipid-lowering effect of TUDCA largely depends on the expression of ADRA2C (supplementary Figure S4A).

To verify whether this process is mediated by the cGAMP-PKG pathway, we further examined the intracellular cGAMP content and the phosphorylation level of the downstream effector molecule VASP. The results showed that PA/OA treatment inhibited the accumulation of cGAMP and the expression of p-VASP; TUDCA treatment could restore the cGAMP level and upregulate VASP phosphorylation, while ADRA2C overexpression reversed this trend again (Fig. 6H-I). We used the selective PKG inhibitor KT-5823. When KT-5823 was used alone, there was no significant change in lipid content compared to the model group. KT-5823 could eliminate the effect of TUDCA on reducing TG and NEFA (supplementary Figure S4B). In summary, TUDCA may exert the effect of improving hepatic cell lipid toxicity injury by down-regulating ADRA2C and subsequently activating the cGAMP-PKG signaling pathway.

### TUDCA improves liver damage and lipid metabolism disorders in mice induced by high-fat diet and high-cholesterol diet

**Fig. 7:**
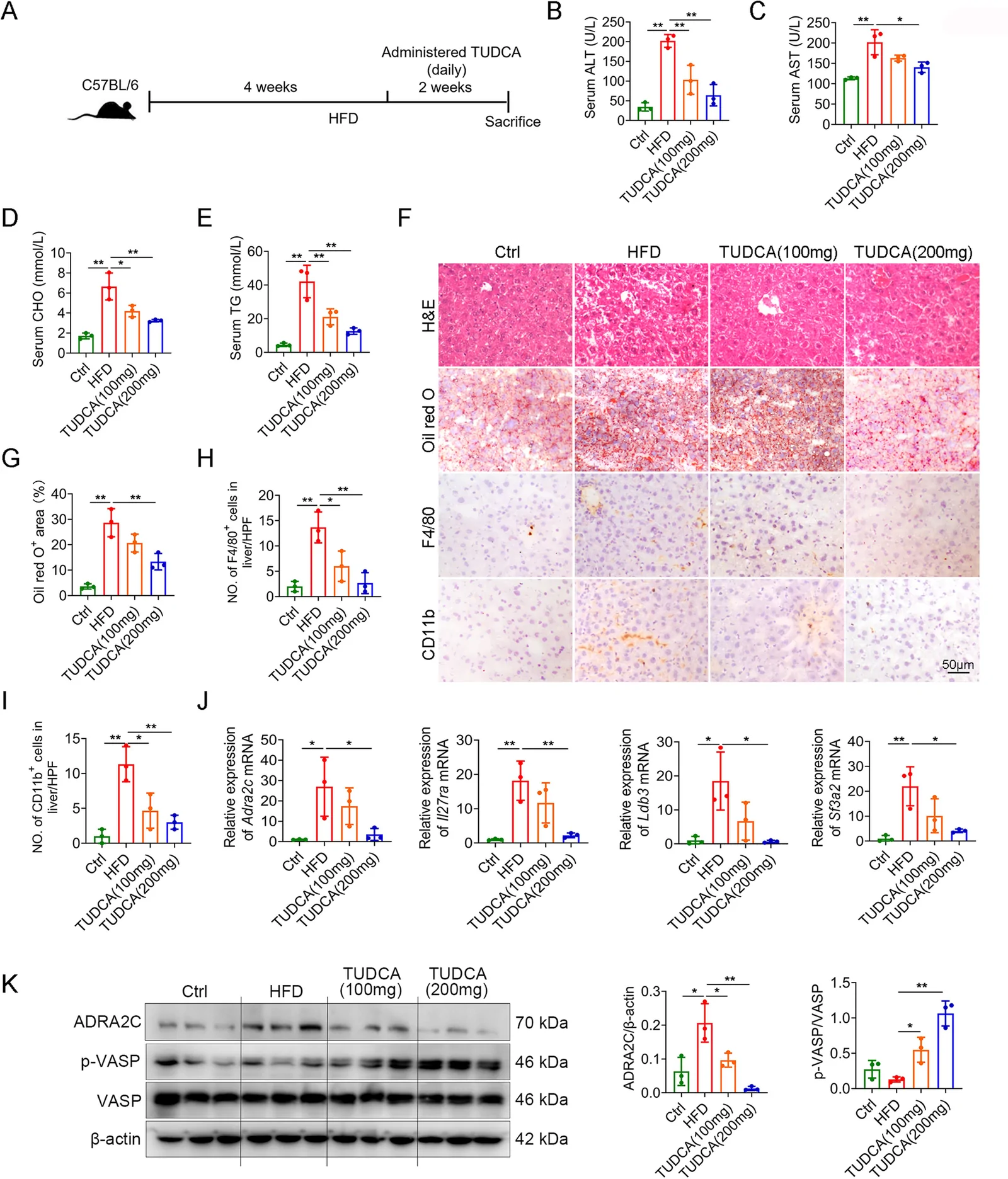
TUDCA alleviated HFD-induced liver injury. C57BL/6 mice (n = 5) were fed HFD and TUDCA treatment. (**A**) Schematic diagram of an HFD-induced model. (**B**-**E**) Serum ALT, AST, CHO and TG levels. (**F**-**I**) Representative H&E, Oil red O, F4/80 and CD11b staining of liver tissues. Statistical analysis of Oil red O, F4/80 and CD11b staining. Scale bar, 50 μm. (**J**) The mRNA levels of Adra2c, Il27ra, Ldb3 and Sf3a2 in liver tissues were measured using RT-qPCR analysis. The results were normalized to Actb. (**K**) The protein expression levels of ADRA2C, VASP and p-VASP in liver tissues were determined using western blot analysis. The densities of protein were quantified using densitometry. *p < 0.05, **p < 0.01

**Fig. 8:**
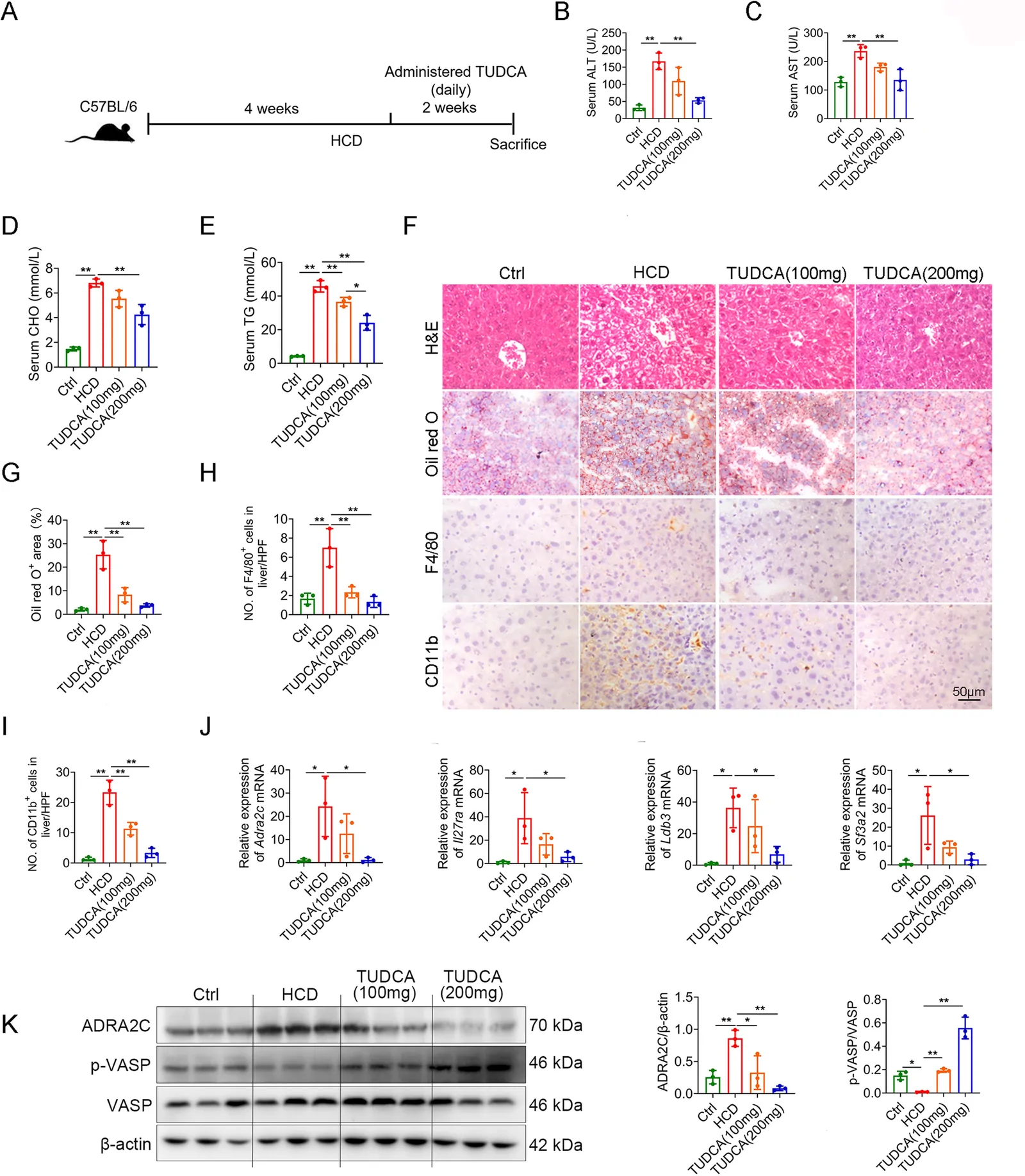
TUDCA alleviated HCD-induced liver injury. C57BL/6 mice (n = 5) were fed HCD and TUDCA treatment. (**A**) Schematic diagram of an HCD-induced model. (**B**-**E**) Serum ALT, AST, CHO and TG levels. (**F**-**I**) Representative H&E, Oil red O, F4/80 and CD11b staining of liver tissues. Statistical analysis of Oil red O, F4/80 and CD11b staining. Scale bar, 50 μm. (**J**) The mRNA levels of Adra2c, Il27ra, Ldb3 and Sf3a2 in liver tissues were measured using RT-qPCR analysis. The results were normalized to Actb. (**K**) The protein expression levels of ADRA2C, VASP and p-VASP in liver tissues were determined using western blot analysis. The densities of protein were quantified using densitometry. *p < 0.05, **p < 0.01

To verify the protective effect of TUDCA at the cellular level, we established two mouse models: one fed with a high-fat diet and the other with a high-cholesterol diet. We administered low-dose (100 mg/kg) and high-dose (200 mg/kg) TUDCA intraperitoneal injections (Figs. 7A and 8A). We monitored food intake and body weight change throughout the experimental period. Compared with the HFD or HCD model groups, the TUDCA treatment group showed no significant differences in weight gain and food intake (supplementary Figure S5). Serum ALT and AST can reflect the degree of liver injury, and serum TG and CHO can be used as diagnostic indicators of lipid metabolism disorders. Serum ALT, AST, CHO, and TG levels were significantly reduced in TUDCA-treated mice compared to HFD-fed and HCD-fed mice (Figs. 7B-E and 8B-E). H&E and Oil red O staining showed that TUDCA treatment could significantly alleviate HFD-induced and HCD-induced hepatic steatosis, and 200 mg TUDCA was the most effective treatment (Figs. 7F and G and 8F and G). In addition, TUDCA treatment significantly reduced inflammatory cell infiltration (F4/80^+^ and CD11b^+^ cells) in HFD-fed and HCD-fed mice by immunohistochemical analysis (Figs. 7F and H-I and 8F and H-I). RT-qPCR analysis of the expression of 4 hub genes revealed that treatment with 200 mg TUDCA significantly reduced the HFD-induced and HCD-induced expression of these genes in the liver (Figs. 7J and 8J). Additionally, western blot analysis revealed that the expression level of ADRA2C in the liver tissues of the two model mice significantly increased, while TUDCA intervention effectively reduced the expression of ADRA2C (Figs. 7K and 8K). The phosphorylation levels of VASP in liver tissues of both model mice were significantly reduced, and TUDCA intervention effectively restored the expression of p-VASP protein (Figs. 7K and 8K). These results indicate that TUDCA can exert protective effects on liver function, reduce lipid accumulation, and alleviate inflammatory responses in both diet-induced metabolic liver injury models.

## Discussion

NASH and FH are two kinds of diseases that seriously affect human health, and there are complex associations between them in the pathophysiological process. Through the combination of bioinformatics analysis and experimental verification, this study conducted a joint study on these two diseases, screened out the common hub genes, and explored the therapeutic role of TUDCA in the two diseases based on molecular docking and animal experiment verification. This study aims to reveal the underlying molecular mechanisms of NASH and FH and screen potential therapeutic targets and drugs.

ADRA2C, IL27RA, LDB3, and SF3A2 were identified as common hub genes with NASH and FH in this study. ADRA2C is a member of the adrenergic receptor family, which plays an important role in regulating metabolism and lipid homeostasis [32, 33]. ADRA2C may influence the progression of NASH by affecting lipid metabolism in the liver [34, 35]. While ADRA2C was selected as the primary focus of this study, the potential contributions of the other three hub genes (IL27RA, LDB3, and SF3A2) to NASH and FH merit consideration. The interleukin-27 receptor encoded by IL27RA plays an important role in immune regulation [36]. Inflammatory response is an important driver of disease progression in NASH, and IL27RA is involved in the inflammatory process in NASH by regulating the function of immune cells and the secretion of inflammatory factors [37]. At the same time, abnormal lipid metabolism triggers immune response in FH patients, IL27RA may play a bridge role in this process, linking lipid metabolism disorders with inflammatory response, and jointly affecting the development of the disease. The protein encoded by the LDB3 gene is abundant in muscle and heart tissue, and also plays a role in the liver [38, 39]. The abnormality of LDB3 may lead to the accumulation of cholesterol in the liver and promote the development of FH by affecting the transport and metabolism of cholesterol by hepatocytes, and may also aggravate the liver damage in NASH patients. SF3A2 is an important protein involved in the RNA splicing process, affecting the mRNA splicing of certain key metabolic enzymes or inflammatory factors, leading to their abnormal expression, thereby promoting the progression of disease [40, 41]. SF3A2 may influence the occurrence and progression of NASH and FH diseases by regulating the splicing of genes associated with lipid metabolism and inflammatory response. Recent studies have shown that molecules located at the intersection of multiple pathways such as inflammation, oxidative stress, and cell death have a stronger pathological regulatory weight, and the same molecule may have completely different functions in different cell types [42–44]. Among the four hub genes, ADRA2C was selected as the main research object because it has the dual functions of regulating lipid metabolism and inflammatory response.

TUDCA has many biological activities, including regulating bile acid metabolism, improving liver lipid metabolism and alleviating inflammation [45]. TUDCA reduces liver inflammation and damage in the treatment of NASH by regulating the composition and metabolism of bile acids, reducing liver fat accumulation, while inhibiting the infiltration of inflammatory cells and the release of inflammatory factors [46, 47]. For patients with FH, TUDCA reduces blood cholesterol levels and improves lipid metabolism disorders by affecting cholesterol metabolism and transport. TUDCA has demonstrated excellent cost-effectiveness in clinical applications. Relevant health technology assessments have confirmed that it is more economical than traditional bile acid drugs in the treatment of specific liver diseases. In terms of safety, current evidence indicates that TUDCA has good tolerance, with the main adverse reactions being mild to moderate gastrointestinal reactions (such as diarrhea and nausea), which can usually resolve on their own. No serious adverse events have been reported, suggesting that it has good feasibility and safety in the exploration of treatment for NASH and FH. TUDCA exerts its therapeutic effect by acting on hub gene-related signaling pathways such as ADRA2C, IL27RA, LDB3 and SF3A2. For example, it may regulate ADRA2C-mediated adrenergic signaling pathways, improve liver fat metabolism, and reduce inflammatory responses by affecting IL27RA-related immunomodulatory pathways. Given the numerous targets present in various liver diseases, ADRA2C possesses several unique characteristics that make it a promising therapeutic strategy: Firstly, unlike many single-pathway targets, ADRA2C regulates both lipid metabolism and inflammatory responses simultaneously. This dual function is particularly important for NASH, as these two metabolic dysfunctions and inflammatory responses often coexist and reinforce each other. This integrated mechanism distinguishes ADRA2C from conventional targets that typically target metabolism or inflammation alone. Secondly, as a G protein-coupled receptor (GPCR) located on the cell surface, ADRA2C is easy to be intervened with drugs. GPCR are the most successful class of drug targets, with approximately 34% of approved drugs targeting this family. The extracellular accessibility of ADRA2C avoids the delivery challenges faced by endogenous targets.

This study conducted a systematic bioinformatics analysis based on the GEO dataset and revealed potential molecular mechanisms, but there are still certain limitations: animal models cannot fully simulate the pathophysiological processes of human NASH and FH (such as genetic heterogeneity of the disease and the influence of environmental factors), and the efficacy of candidate drugs in animals may not be fully replicated in humans. In the transcriptional regulation analysis, only the relevant motifs were pointed out, and the direct binding and regulatory effects of the transcription factors corresponding to this motif to the hub genes were not deeply verified. The failure to verify the expression pattern of the hub gene in patient samples (whether it is abnormal in both NASH and FH patients) has weakened the direct evidence for the clinical translation of the research results. Recent studies underscore the importance of parenchymal-non-parenchymal crosstalk: hepatocyte-derived exosomes mediate intercellular signaling in liver diseases, hepatocyte lipid accumulation alters immune surveillance and promotes inflammatory macrophage polarization, and hepatic stellate cells transfer fatty acids and mitochondria to other cells via MTFR2-driven mitochondrial fission [48–50]. This study only utilized hepatocytes (the parenchymal cells) and did not investigate the non-parenchymal cell population, particularly hepatic macrophages (Kupffer cells). Considering the significant role of macrophages in liver inflammation, fibrosis, and metabolic regulation, this limitation is particularly notable. Recent studies have shown that macrophages play a critical role in liver inflammation, fibrosis, and metabolic regulation [42, 51]. Since non-parenchymal cells account for approximately 30% to 40% of the liver cell composition, excluding these cells would limit our understanding of the functional role of ADRA2C in the complex liver microenvironment. To improve the translational potential of this study, future studies will use clinical samples and combine multi-omics data (such as single-cell sequencing, spatial transcriptome) to deeply analyze the spatio-temporal specificity of molecular mechanisms. This study provides important theoretical basis and candidate targets for subsequent work, and exploring their heterogeneity in different diseases is conducive to ultimately promoting the application of precision medicine.

## Conclusion

In conclusion, we identified hub genes such as ADRA2C, IL27RA, LDB3 and SF3A2 as well as TUDCA as drug candidate through joint analysis of NASH and FH diseases. It provides an important theoretical basis and research direction for the in-depth understanding of the pathogenesis of these two diseases and the development of new therapeutic strategies.

## Supplementary Information

Below is the link to the electronic supplementary material.

Supplementary Material 1

Supplementary Material 2

## Data Availability

The original contributions presented in the study are included in the article/Supplementary Material. Further inquiries can be directed to the corresponding authors.
