# Supplementary material for: TUDCA was identified as a potential therapeutic drug for nonalcoholic steatohepatitis complicated with hypercholesterolemia targeting ADRA2C by integrated bioinformatics and experimental verification
Source: Biol Direct. 2026 Aug 8;21:227. doi: 10.1186/s13062-026-00926-w (PMC13737800; doi:10.1186/s13062-026-00926-w)

Figure 5. (C) The protein expression level of ADRA2C and β-actin

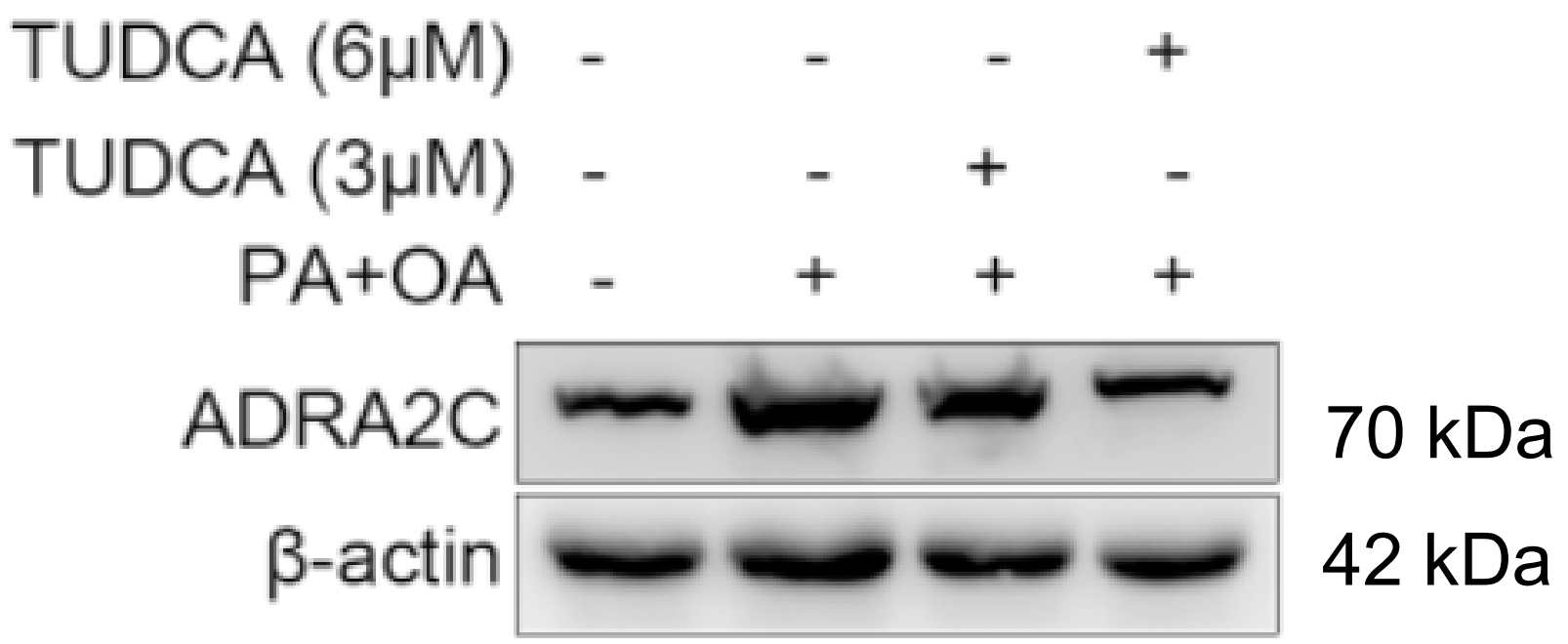

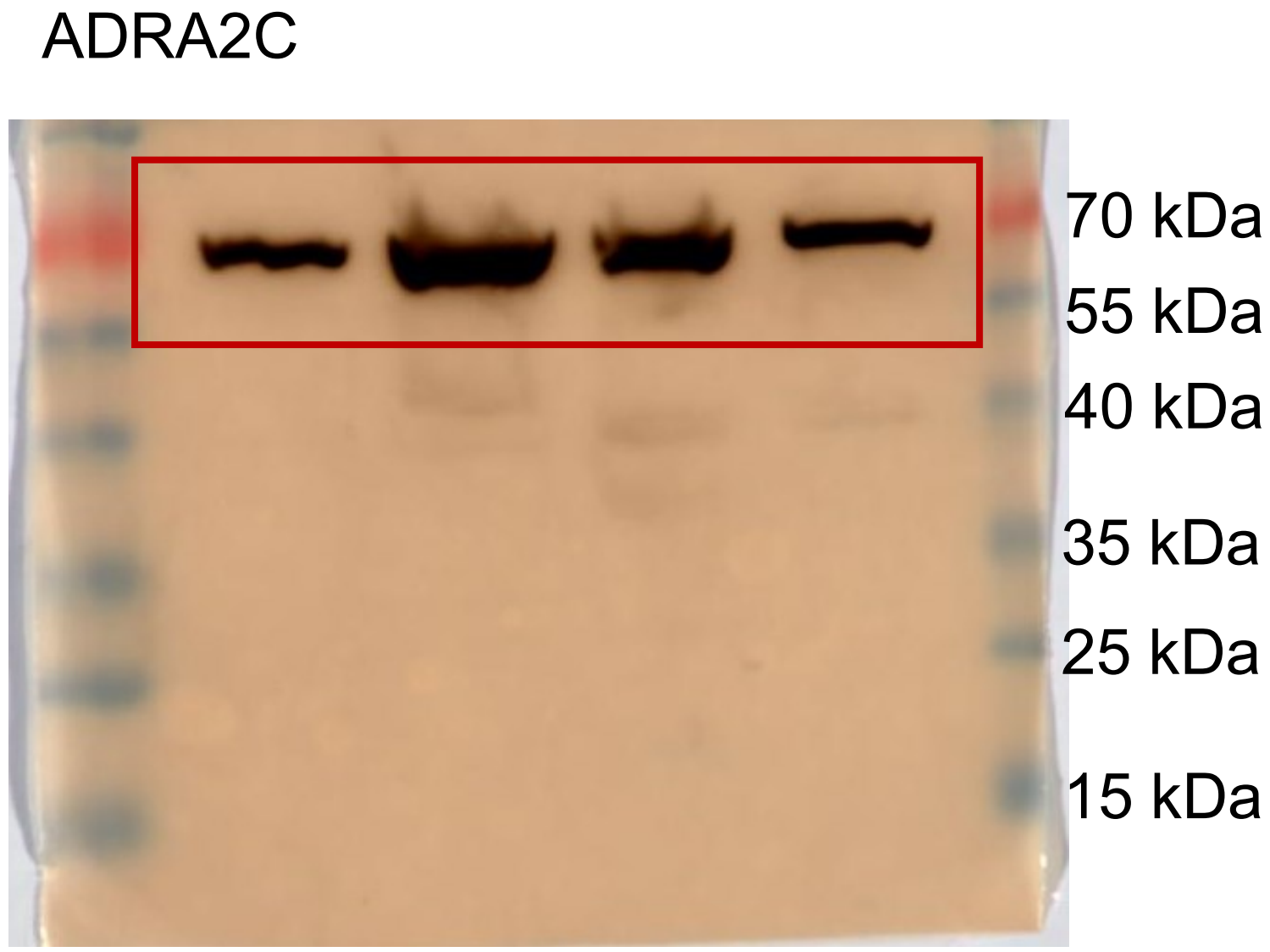

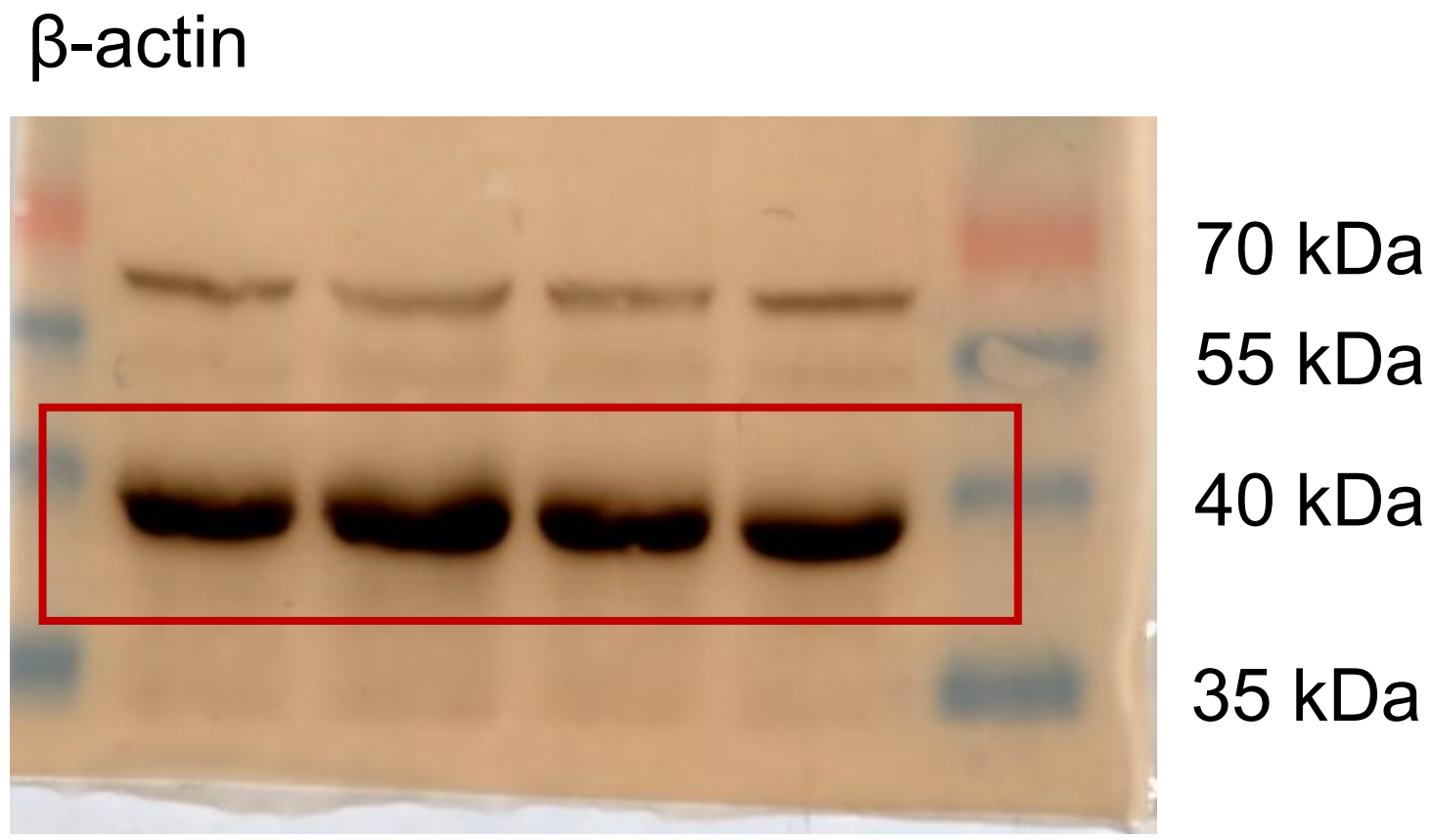

Figure 5. (D) The protein expression level of ADRA2C and β-actin

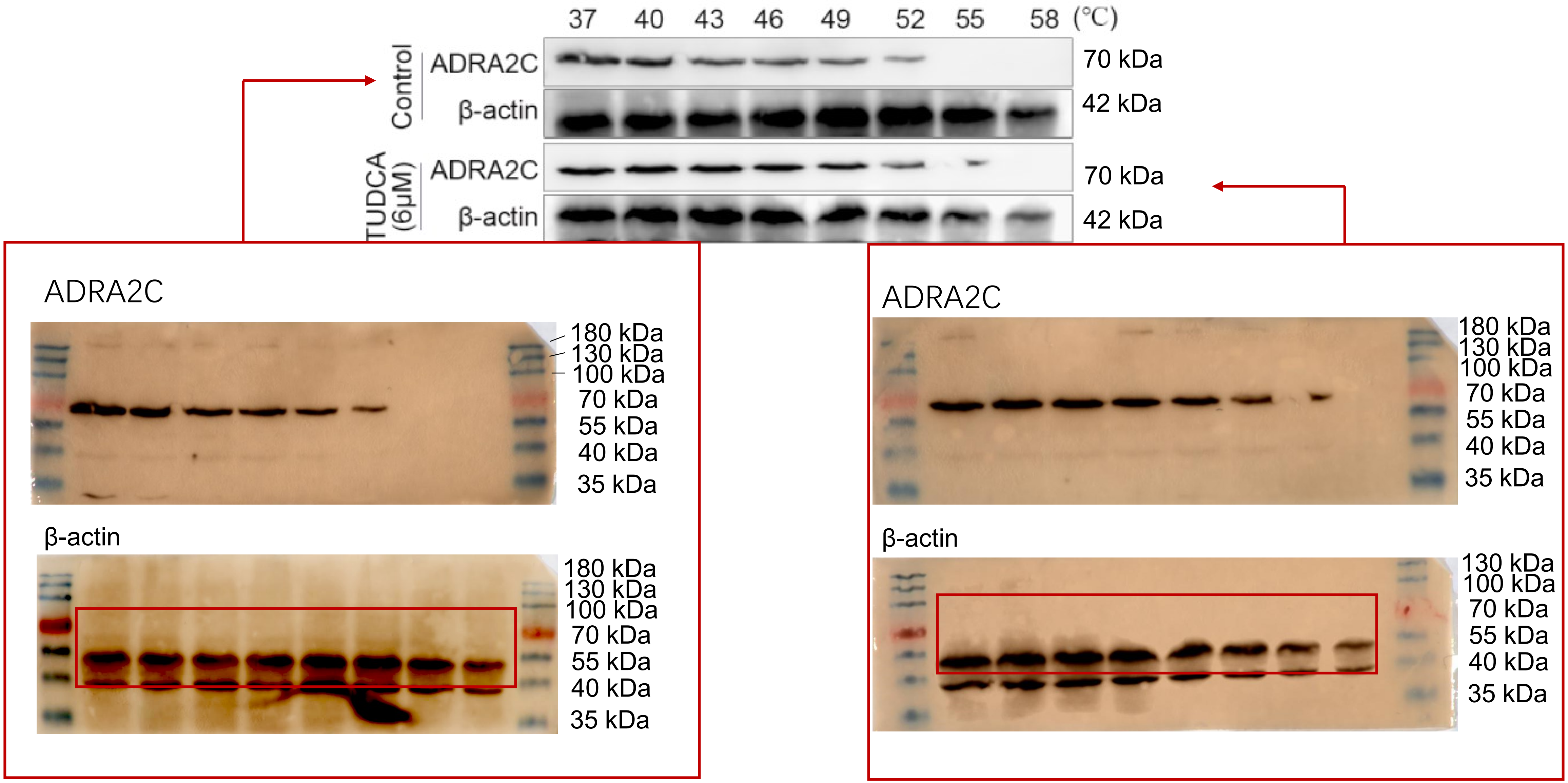

Figure 6. (C) The protein expression level of ADRA2C and β-actin

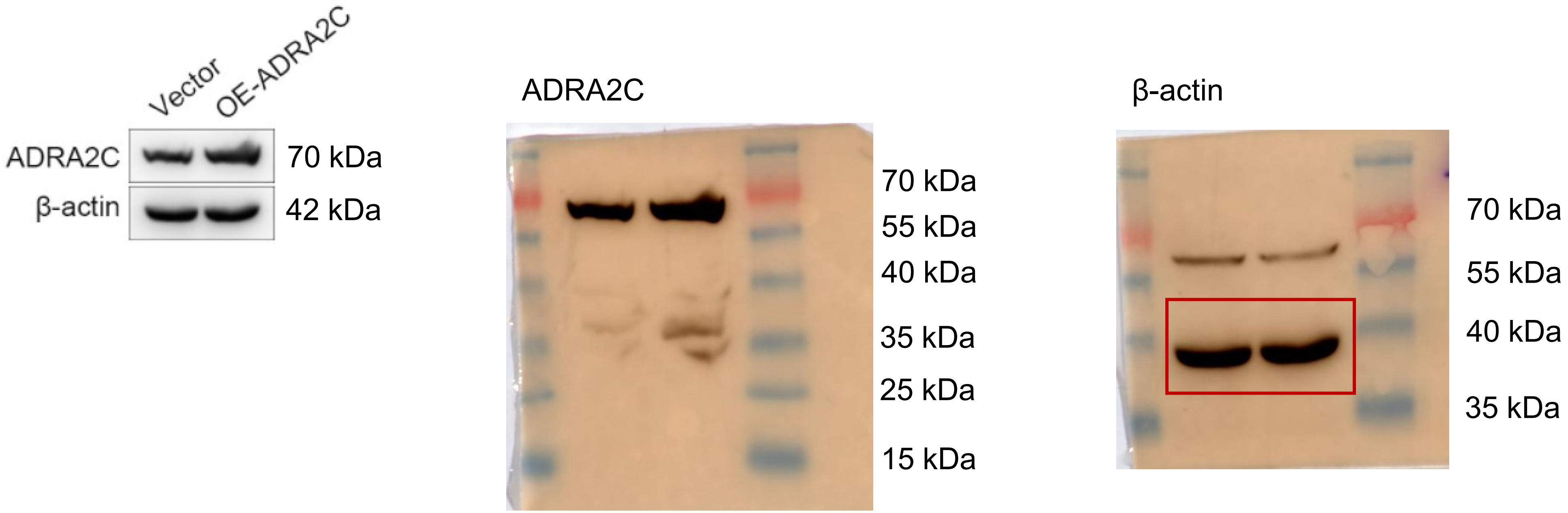

Figure 6. (I) The protein expression level of VASP and β-actin

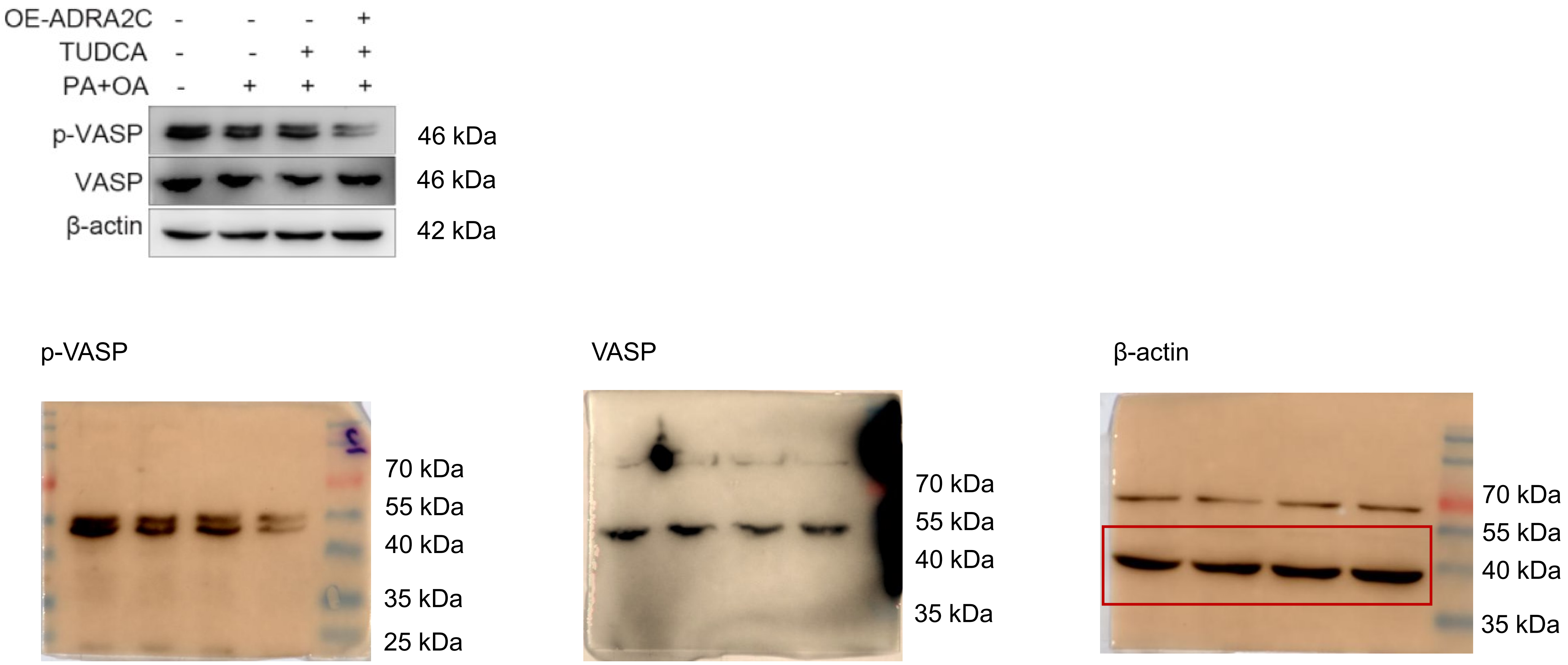

Figure 7. (K) The protein expression level of VASP and β-actin

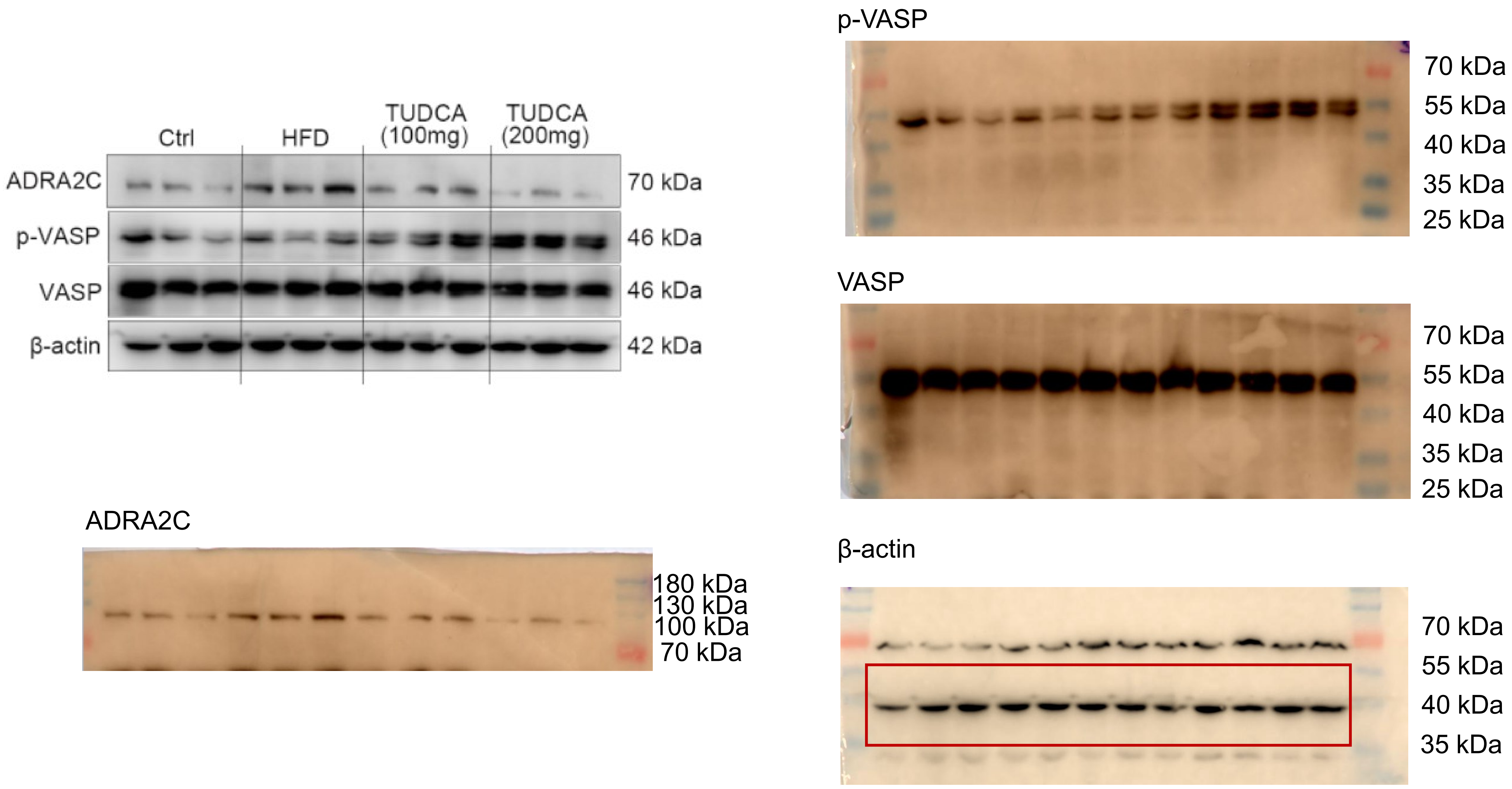

Figure 8. (K) The protein expression level of VASP and β-actin

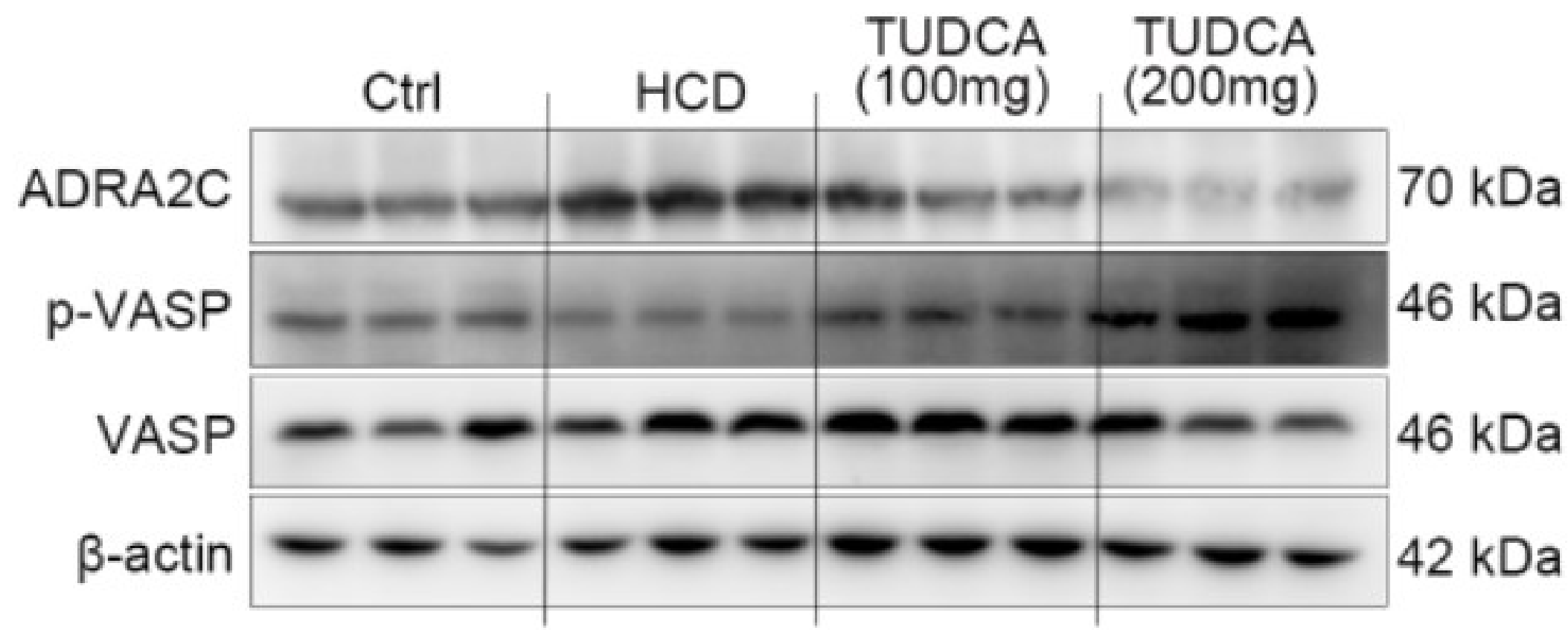

ADRA2C

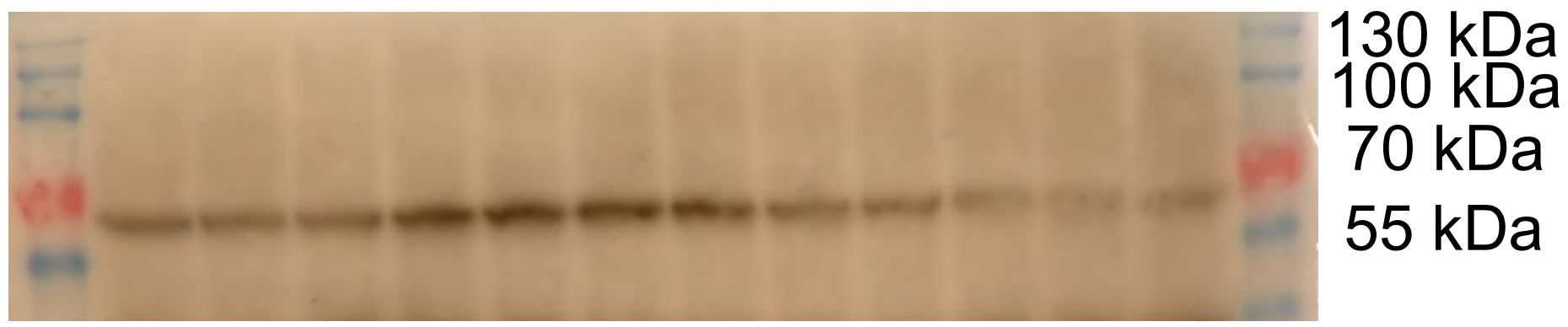

p-VASP

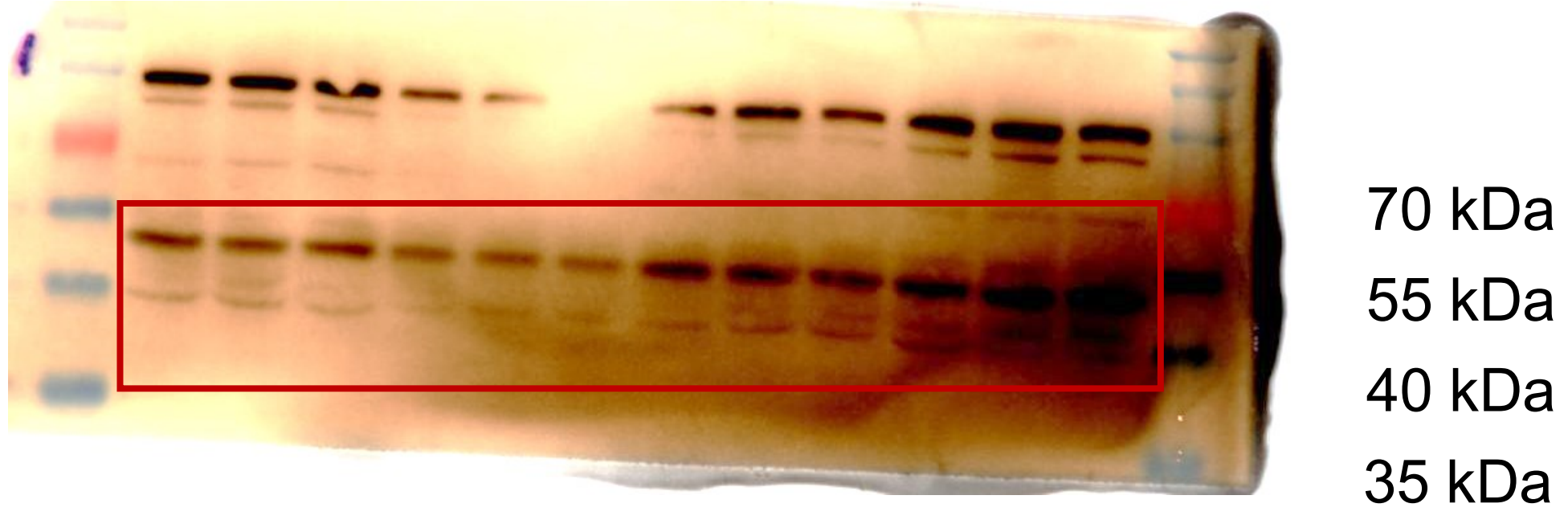

VASP

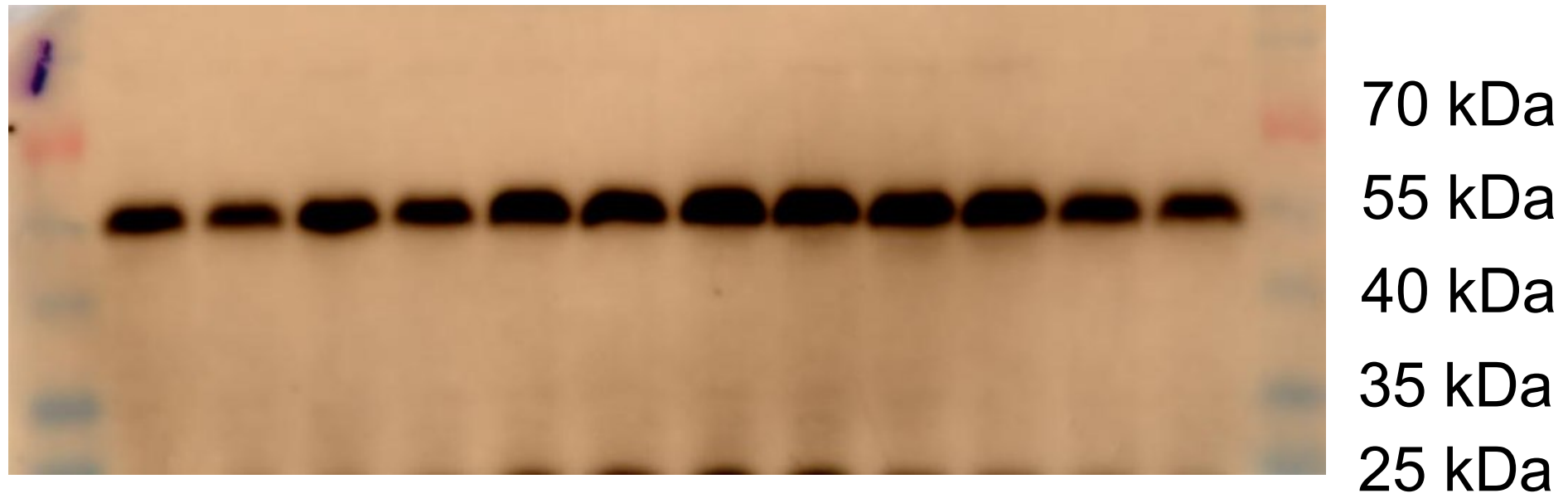

β-actin

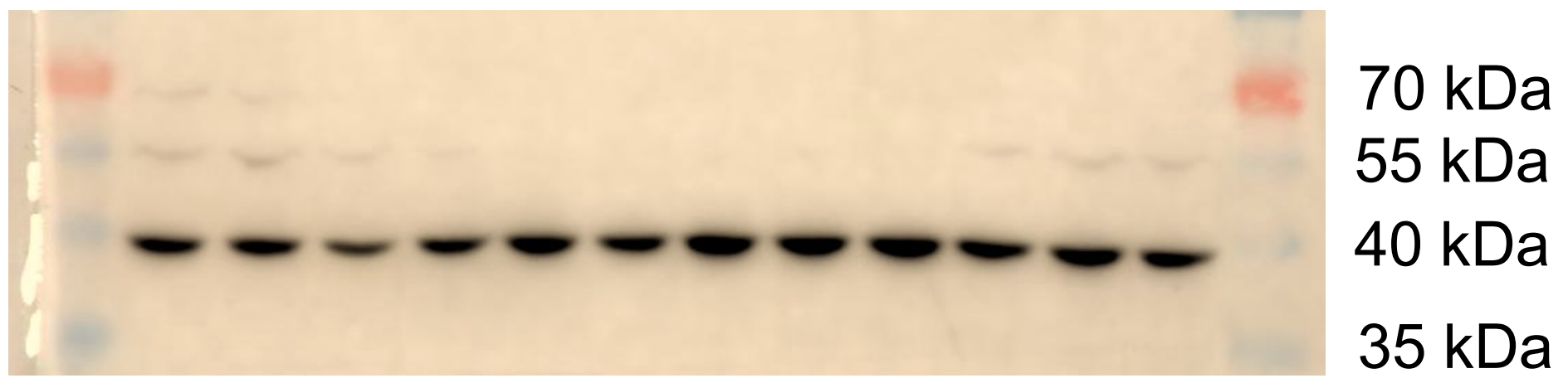

Supplement: Supplementary file 1 [file 13062_2026_926_MOESM2_ESM.pdf]
