# Supplementary material for: TUDCA was identified as a potential therapeutic drug for nonalcoholic steatohepatitis complicated with hypercholesterolemia targeting ADRA2C by integrated bioinformatics and experimental verification
Source: Biol Direct. 2026 Aug 8;21:227. doi: 10.1186/s13062-026-00926-w (PMC13737800; doi:10.1186/s13062-026-00926-w)
Supplement: Supplementary file 2 [file 13062_2026_926_MOESM1_ESM.docx]

Figure S1. Data Preparation.

(A, B) Boxplots of expression genes before and after normalization of GSE37031 and GSE59045. (C, D) Boxplots of expression genes before and after normalization of GSE6054 and GSE13985.

Figure S2. Demonstration of the highest motif enrichment of AUC, including NES, AUC, and enrichedGenes.

Figure S3. Molecular docking analysis of lycopene with ADRA2C, IL27RA, LDB3, and SF3A2.

Figure S4. ADRA2C mediates TUDCA-induced lipid-lowering effects through PKG signaling. (A) Hepatocytes were pretreated with ADRA2C siRNA to PA+OA and/or TUDCA treatment. Cellular TG and NEFA levels were measured. (B) Hepatocytes were pretreated with KT-5823 (1 μM, selective PKG inhibitor) for 30 min prior to PA+OA and/or TUDCA treatment. Cellular TG and NEFA levels were measured. **P < 0.01.

Figure S5. Body weight gain and daily food intake.

(A) Body weight gain and average daily food intake in HFD-fed mice. (B) Body weight gain and average daily food intake in HCD-fed mice.
